# Stressors, coping, and resources needed during the COVID-19 pandemic in a sample of perinatal women

**DOI:** 10.1186/s12884-021-03665-0

**Published:** 2021-03-01

**Authors:** Celestina Barbosa-Leiker, Crystal Lederhos Smith, Erica J. Crespi, Olivia Brooks, Ekaterina Burduli, Samantha Ranjo, Cara L. Carty, Luciana E. Hebert, Sara F. Waters, Maria A. Gartstein

**Affiliations:** 1 College of Nursing, Washington State University Health Sciences Spokane, 412 E. Spokane Falls Blvd, Spokane, WA 99202-2131 USA; 2 Elson S. Floyd College of Medicine, Washington State University Health Sciences Spokane, Spokane, WA USA; 3 School of Biological Sciences and Center for Reproductive Biology, Washington State University, Pullman, WA USA; 4 Washington State University Health Sciences Spokane, Institute for Research and Education to Advance Community Health, Seattle, WA USA; 5 Department of Human Development, Washington State University, Vancouver, WA USA; 6 Department of Psychology, Washington State University, Pullman, WA USA

**Keywords:** COVID-19, Perinatal, Pregnancy, Postpartum, Parenting, Stress, Social support, Coping, Health disparities

## Abstract

**Background:**

Psychological stress and coping experienced during pregnancy can have important effects on maternal and infant health, which can also vary by race, ethnicity, and socioeconomic status. Therefore, we assessed stressors, coping behaviors, and resources needed in relation to the COVID-19 pandemic in a sample of 162 perinatal (125 pregnant and 37 postpartum) women in the United States.

**Methods:**

A mixed-methods study captured quantitative responses regarding stressors and coping, along with qualitative responses to open-ended questions regarding stress and resources needed during the COVID-19 pandemic. Logistic and linear regression models were used to analyze differences between pregnant and postpartum participants, as well as differences across key demographic variables. Qualitative content analysis was used to analyze open-ended questions.

**Results:**

During the COVID-pandemic, food scarcity and shelter-in-place restrictions made it difficult for pregnant women to find healthy foods. Participants also reported missing prenatal appointments, though many reported using telemedicine to obtain these services. Financial issues were prevalent in our sample and participants had difficulty obtaining childcare. After controlling for demographic variables, pregnant women were less likely to engage in healthy stress-coping behaviors than postpartum women. Lastly, we were able to detect signals of increased stressors induced by the COVID-19 pandemic, and less social support, in perinatal women of racial and ethnic minority, and lower-income status. Qualitative results support our survey findings as participants expressed concerns about their baby contracting COVID-19 while in the hospital, significant others missing the delivery or key obstetric appointments, and wanting support from friends, family, and birthing classes. Financial resources, COVID-19 information and research as it relates to maternal-infant health outcomes, access to safe healthcare, and access to baby supplies (formula, diapers, etc.) emerged as the primary resources needed by participants.

**Conclusions:**

To better support perinatal women’s mental health during the COVID-19 pandemic, healthcare providers should engage in conversations regarding access to resources needed to care for newborns, refer patients to counseling services (which can be delivered online/via telephone) and virtual support groups, and consistently screen pregnant women for stressors.

**Supplementary Information:**

The online version contains supplementary material available at 10.1186/s12884-021-03665-0.

## Background

Since the coronavirus disease 2019 (COVID-19) pandemic began, perinatal women have experienced psychological stress due to changes in labor and delivery hospital policies [1], possible perinatal COVID-19 transmission [2], and COVID-19 clinical maternal-infant outcomes [3]. These perinatal-related stressors are in addition to the economic and mental health issues that many people are currently experiencing. While stressors for perinatal women can be found worldwide, pregnant women in the United States have the highest maternal mortality rate in the developed world, and social and environmental stressors contribute to a woman’s risk of dying within 1 year of pregnancy [4]. Our previous research demonstrated higher levels of anxiety and depression in American pregnant women compared to Dutch [5] pregnant women and higher levels of psychological stress compared to British pregnant women [6].

Psychological stress experienced during pregnancy can have deleterious effects on maternal and infant health [7], where the pathway between stress and physiological issues involves cortisol, norepinephrine, and inflammatory markers, including cytokines [7, 8]. The health ramifications due to perinatal stress not only impact the mother but also may harm the fetus and infant. For example, elevated glucocorticoids during fetal development are associated with a higher likelihood of adverse birth outcomes (e.g., pre-term birth and intrauterine growth restriction) and predisposition to obesity and other late-onset diseases [9]. In addition, effects of prenatal stress on offspring neurodevelopment, cognitive development, negative affectivity, difficult temperament, and psychiatric disorders have been demonstrated in numerous epidemiological and case-control studies [10]. The American College of Obstetricians and Gynecologists has acknowledged the impact of psychological stress on infant and maternal health and therefore recommends perinatal screening and intervention for stress for all pregnant women [11].

The assessment of coping is crucial to understand the ways in which psychological stress and stressful life events can be buffered [12–15], with the adaptative value of coping stemming from being able to control the stressor or relying on support from others (i.e., social support). In addition, sociocultural contexts have to be considered in the study of perinatal stress and coping [16] as effects of psychosocial stress experienced during pregnancy can also vary by race, ethnicity, and socioeconomic status [17, 18]. For example, there are increased incidences of adverse birth outcomes, including higher infant mortality rates, fetal mortality rates, and preterm birth, in African American/Black and Latino women compared to European Americans [19–21]. In addition, women in lower socioeconomic groups have an increased risk for stress and pregnancy complications that are independent of other factors [22].

While research on stress and coping of perinatal women during the COVID-19 pandemic has yet to be published, reports on pregnant women in China indicate that the COVID-19 pandemic may increase the risk for mental illness [23] and research with pregnant women in the United States demonstrate elevated levels of anxiety and stress [24], particularly in vulnerable groups (i.e., primiparas, women with high risk or unplanned pregnancy, survivors of abuse, and women of color) [25]. Therefore, we assessed stressors, coping behaviors, and resources needed in relation to the COVID-19 pandemic in a sample of perinatal women in the United States using a concurrent mixed methods study design.

## Methods

### Participants

Using social media and a crowdsourcing platform, Prolific (www.prolific.co), women were directed to online surveys from 4/28/2020–6/30/2020. Inclusion criteria were ≥ 18 years of age, English-speaking, currently pregnant or postpartum (given birth between 9/1/2019–5/31/2020). While “perinatal” is often used to describe pregnancy at about the 20th week of gestation through approximately 1–4 weeks postpartum, we use the terms “perinatal” and “postpartum” that adhere to the timeframe relevant to perinatal depression, which includes pregnancy through 12 months post-delivery [26] Those who met inclusion criteria (*N* = 228) were presented with an online informed consent form, which they could download or email to themselves. One hundred sixty-two people (77% completion rate; *n* = 79 participants from social media recruitment and *n* = 83 participants from the crowdsourcing platform) consented to participate by clicking a statement indicating that they had reviewed the consent form and agreed to participate. Eligible participants also were provided a list of investigators within the consent form which detailed their name, credentials, contact information, and study role.

Utilizing a subset of perinatal participants (*n* = 79) (i.e., participants from the social media recruitment strategy), we asked additional questions related to social support and open-ended questions regarding stressors and resources needed; these additional items were embedded within the online survey.

### Measures

Demographic information included age, race, ethnicity, pregnancy status, and location of residence. Health insurance, employment, income, and education information was also collected as informed by the CoRonavIruS Health Impact Survey V0.1 Adult Self-Report Baseline Form [27].

Survey items were selected from the Centers for Disease Control and Prevention COVID-19 Community Survey Question Bank Form [28]. General impacts of COVID-19, including financial and other areas of stress, were measured using multiple selection items. Women were asked, “To cope with social distancing, isolation, or stress related to COVID-19, are you doing any of the following?” and were provided a list of coping behaviors (e.g., taking care of your body, eating healthy/unhealthy foods, exercising more/less, connecting with others, taking breaks from media, making time to relax, substance use). Women were also asked to report recent (within the past 2 weeks) financial impacts, including serious financial problems, income/pay reduction, being laid off or furloughed by employer, having a household member lose their job, etc.

Survey items pertaining to COVID-19 diagnosis and medical history, were informed by the CoRonavIruS Health Impact Survey V0.1 Adult Self-Report Baseline Form [27]. Women were asked (yes/no) if they had received a COVID-19 diagnosis for self, family member(s) inside household, or family member(s) outside household. Pregnancy and postpartum information were also collected. Changes to prenatal healthcare behaviors due to COVID-19 were measured using a checklist of common experiences (e.g., missed prenatal appointments, using telemedicine for prenatal appointments, and talking to their provider about labor, delivery, and COVID-19). Women were also asked to select reasons for difficulty in obtaining healthy foods to support pregnancy: financial hardship, scarcity, and shelter in place restrictions. Items relating to pregnancy-specific behaviors were given only to the pregnant participants as postpartum participants could have entered the study pregnant pre-pandemic and postpartum only during the pandemic.

A subset of perinatal women responded to three open-ended questions regarding stressors and resources needed during the COVID-19 pandemic. Social support was then assessed using the Interpersonal Support Evaluation List (ISEL) Short Form, a 12-item measure of three dimensions: Appraisal Support (perceived availability of someone to talk to about one’s problems); Belonging Support (perceived availability of people one can do things with); and Tangible Support (perceived availability of material aid) [29]. Each dimension is measured by four items on a 4-point scale ranging from “Definitely True” to “Definitely False” with higher scores indicating more social support. Scores on the total ISEL and each subscale were normally distributed based on skewness and kurtosis statistics. Each ISEL subscale was statistically significantly related to the total ISEL score (total ISEL with Appraisal Support *r* = .86, *p* < .01; total ISEL with Belonging Support *r* = .66, *p* < .01; total ISEL with Tangible Support *r* = .67, *p* < .01). Cronbach’s alpha indicated that the total ISEL score (.91) and each subscale (Appraisal Support = .81; Belonging Support = .83; Tangible Support = .80) was internally consistent for our perinatal sample. Please refer to the Additional file 1 for the open-ended qualitative questions and survey items.

### Study design

A concurrent mixed-methods study design, using cluster sampling of perinatal women in the United States, was used to capture quantitative responses on stress- and coping-related survey items and qualitative responses to open-ended questions regarding stressors and resources needed during the COVID-19 pandemic. Both types of responses were analyzed concurrently, and data was mixed after quantitative and qualitative analyses were completed. Capturing both survey and qualitative responses provided a comprehensive assessment of stressors, coping, and needs of perinatal women during the COVID-19 pandemic. Please refer to the Supplementary Table for the Consolidated criteria for reporting qualitative studies (COREQ): 32-item checklist. The survey took participants 10 min on average to complete. The ISEL and three open-ended questions took participants and additional 15 min on average to complete. This study was deemed exempt by Washington State University’s Institutional Review Board.

### Statistical analysis

Descriptive statistics are reported for pregnant and postpartum women separately. Mean (M) and standard deviations (SD) are presented for continuous variables and the percent of participants endorsing each category is reported for categorical variables.

Logistic regression (for binary outcomes) and linear regression (for continuous outcomes) were used to compare responses across pregnant vs. postpartum women (0 = postpartum; 1 = pregnant), controlling for age (continuous), income (0 = less than $25,000–$74,999; 1 = $75,000 and higher), state/federally-funded insurance (0 = self, employer, military, other, or no insurance; 1 = Medicaid, Medicare/CHP), and race/ethnicity (0 = Hispanic, Black, Asian, multiple race; 1 = non-Hispanic White). Odds ratios (OR), 95% confidence intervals (CI), and *p*-values are reported for logistic regression results and standardized regression coefficients (*b*) and p-values are reported for linear regression results. *P* ≤ .05 was used to indicate statistical significance. All analyses were conducted in SPSS version 26.

### Content analysis

Qualitative content analysis [30] using an inductive approach was used to analyze responses to the following open-ended questions: What are you most worried about regarding COVID-19 and your pregnancy, or if you have already delivered your baby, what are you most worried about regarding COVID-19 and newly parenting? Are there things that you are lacking (food, diapers, etc.) that are making you feel stressed or anxious? What resources would be most helpful to you?

Field notes were taken at coder analytic meetings and at the various iterations of coding conducted to reduce the data into themes. For each response, themes were generated for the words and phrases expressed by the respondents (implicit and explicit). Themes were then coded using a set of mutually exclusive categories (i.e., concepts). We then assessed frequency of each concept. Conceptual content analysis was completed by two coders and then verified by a third coder. Any discrepancies in theme development, list of concepts, or concept coding were discussed among the coders and the final coding was reached via consensus. Data saturation was discussed with the coding team. Upon first meeting to discuss data coding, all coders agreed that saturation was reached based on frequency of consistent data supporting each theme, with no new data offering additional unique information. Qualitative data was managed in SPSS version 26.

## Results

### Participant characteristics

Participants were 162 pregnant or postpartum women (125 pregnant and 37 postpartum; gave birth between 9/1/2019–5/31/2020) women aged 19–45 years (Mean = 31, SD = 4.8); 79% non-Hispanic White, 7% Hispanic/Latino, 5% Black, 4% Asian, and 5% more than one race. Ninety-six percent of the sample were employed, homemaker, or a student, 96% were covered by health insurance, 19% had state/federally-funded insurance, and 56% had a total household income ≥$75,000/year. Thirty-nine percent of pregnant women had no children in their household, with the remaining participants reporting 1–4 children in their household (M = .99, SD = .98); postpartum women had a range of 1–5 children in their household (M = 1.65, SD = .95). No participants were positive for COVD-19; one participant had a family member test positive for COVID-19.

The subset of perinatal participants that was given additional items related to social support, stressors, and resources needed included 79 women (42 pregnant and 37 postpartum) women aged 23–42 years (Mean = 32, SD = 4.0); 88% non-Hispanic White, 8% Hispanic/Latino, 1% Asian, and 3% more than one race. All participants in the subsample were employed, homemaker, or a student, 99% were covered by health insurance, 8% had state/federally-funded insurance, and 72% had a total household income ≥$75,000/year. Fifty-seven percent of pregnant women had no children in their household, with the remaining participants reporting 1–3 children in their household (M = .64, SD = .85); postpartum women had a range of 1–5 children in their household (M = 1.65, SD = .85).

### Prevalence of stressors and coping behaviors

Please refer to Table 1 for prevalence of individual stressors and coping behaviors reported by perinatal participants. During the COVID-19 pandemic, 27% of pregnant women reported inability to obtain healthy food. Sixteen percent of pregnant participants reported that shelter-in-place restrictions impacted their access to healthy foods whereas 7% reported financial hardship as the reason they were unable to obtain healthy foods.

**Table 1:** Prevalence of stressors and health behaviors in perinatal women during the COVID-19 pandemic (*N* = 162)

	Pregnant *N* = 125 N (%)	Postpartum *N* = 37 N (%)
**Since the onset of the COVID-19 pandemic, have you been unable to purchase healthy foods to support your pregnancy due to:**		
financial hardship	9 (7%)	N/A
scarcity in stores	34 (27%)	N/A
shelter in place restrictions	20 (16%)	N/A
**Since the onset of the COVID-19 pandemic, have you experienced any of the following?**		
Missed any prenatal care appointments due to COVID-19	39 (31%)	2 (5%)
Used telemedicine for any prenatal care appointments due to COVID-19	51 (41%)	7 (19%)
Sought additional information about how COVID-19 is impacting the hospital you plan to deliver	60 (48%)	14 (38%)
Talked to your provider about labor and delivery and COVID-19	64 (51%)	15 (40%)
**How has the COVID-19 outbreak affected you in the past 2 weeks?**		
Worked remotely or from home more than you usually do	49 (39%)	11 (30%)
Worked more hours than usual	17 (14%)	0 (0%)
Worked reduced hours	18 (14%)	5 (14%)
Was not able to work	19 (15%)	2 (5%)
Had difficulty arranging for childcare	22 (18%)	9 (24%)
Incurred increased costs for childcare expenses	3 (2%)	0 (0%)
Income or pay has been reduced	26 (21%)	5 (14%)
Have no income	3 (2%)	1 (3%)
Was laid off or furloughed by employer	12 (10%)	2 (5%)
Had serious financial problems	14 (11%)	1 (3%)
Another household member has lost their job	15 (12%)	2 (5%)
**In the past two weeks have you experienced the following as a result of COVID-19?**		
Not enough money to pay rent	14 (11%)	0 (0%)
Not enough money to pay for gas	9 (7%)	0 (0%)
Not enough money to pay for food	9 (7%)	0 (0%)
Did not have a regular place to sleep or stay	2 (2%)	0 (0%)
**To cope with social distancing, isolation, or stress related to COVID-19, are you doing any of the following?**		
Taking breaks from watching, reading, or listening to news stories, including social media	84 (67%)	27 (73%)
Taking care of your body, such as taking deep breaths, stretching, or meditating	62 (50%)	10 (27%)
Engaging in healthy behaviors like trying to eat healthy, well-balanced meals, exercising regularly, getting plenty of sleep, or avoiding alcohol and drugs	80 (64%)	21 (57%)
Making time to relax	78 (62%)	18 (49%)
Connecting with others, including talking with people you trust about your concerns and how you are feeling	68 (54%)	21 (57%)
Contacting a healthcare provider	20 (16%)	7 (19%)
Using sleeping medications or sedatives/hypnotics	4 (3%)	0 (0%)
Eating high fat or sugary foods	49 (39%)	16 (43%)
Eating more food than usual	39 (31%)	10 (27%)
Eating less food than usual	13 (10%)	2 (5%)

Note. N/A denotes not applicable as only pregnant women received those survey questions

While 25% (31% pregnant and 5% postpartum) of the participants missed prenatal appointments, 36% (41% pregnant and 19% postpartum) reported using telemedicine for prenatal appointments and about half talked to their provider about labor and delivery and COVID-19 (49% total; 51% pregnant and 40% postpartum). Forty-six percent of the sample sought additional information about how COVID-19 was impacting the hospital where they planned to deliver (48% pregnant) or had delivered (38% postpartum).

In terms of financial stressors, 19% of participants (21% pregnant and 14% postpartum) had income/pay reduced, 9% (10% pregnant and 5% postpartum) were laid off/furloughed, and 10% (12% pregnant and 5% postpartum) had another household member lose a job. Ten percent reported serious financial problems (11% pregnant and 3% postpartum) and 19% (18% pregnant and 24% postpartum) had difficulty arranging childcare.

To cope with social distancing, isolation, or stress related to the COVID-19 pandemic, 69% (67% pregnant and 73% postpartum) took breaks from news outlets and 62% (64% pregnant and 57% postpartum) engaged in healthy behaviors like trying to eat healthy, exercising, getting plenty of sleep, and avoiding alcohol and drugs. Fifty-five percent of participants (54% pregnant and 57% postpartum) connected with others to cope with social distancing, isolation, and stress during the pandemic and 59% (62% pregnant and 49% postpartum) made time to relax.

### Regression analyses

#### Pregnancy and parenthood differences

After accounting for demographic covariates, pregnant women were less likely to engage in healthy stress-coping behaviors than postpartum women, such as taking breaks from watching the news (OR = 0.15, 95%CI = 0.06–0.35), trying to eat healthy, exercise, etc., (OR = 0.32, 95%CI = 0.14–0.72), making time to relax (OR = 0.39, 95%CI = 0.17–0.90), and connecting with others (OR = 0.30, 95%CI = 0.13–0.68). The results are found in Table 2.

**Table 2:** Comparison of stressors and health behaviors in pregnant vs. postpartum women during the COVID-19 pandemic (*N* = 162)

	Odds Ratio (95% CI) *P*-value	Statistically significant covariates
**Since the onset of the COVID-19 pandemic, have you experienced any of the following?** ***(select all that apply)***		
Missed any prenatal care appointments due to COVID-19	7.74 (1.75–34.30) *P* = .007	
Used telemedicine for any prenatal care appointments due to COVID-19	3.91 (1.48–10.37) *P* = .006	
Sought additional information about how COVID-19 is impacting the hospital you plan to deliver	1.69 (0.76–3.77) *P* = .202	
Talked to your provider about labor and delivery and COVID-19	1.76 (0.80–3.89) *P* = .161	
**How has the COVID-19 outbreak affected you in the past 2 weeks?** ***(select all that apply)***		
Worked remotely or from home more than you usually do	1.82 (0.78–4.25) *P* = .166	
Worked reduced hours	1.35 (0.41–4.39) *P* = .619	
Was not able to work	2.34 (0.48–11.28) *P* = .291	Lower income 0.25 (0.08–0.82) *P* = .022
Had difficulty arranging for childcare	0.63 (0.25–1.58) *P* = .321	
Income or pay has been reduced	1.83 (0.58–5.81) *P* = .304	
Have no income	4.07 (0.50–33.30) *P* = .191	
Was laid off or furloughed by employer	1.51 (0.31–7.45) *P* = .612	
Had serious financial problems	2.85 (0.33–24.40) *P* = .339	Lower income 0.20 (0.05–0.87) *P* = .032 Women of color^a^ 0.27 (0.08–0.93) *P* = .038
Another household member has lost their job	5.12 (0.64–40.85) *P* = .123	
**To cope with social distancing, isolation, or stress related to COVID-19, are you doing any of the following?** ***(select all that apply)***		
Taking breaks from watching, reading, or listening to news stories, including social media	0.15 (0.06–0.35) *P* < .001	
Taking care of your body, such as taking deep breaths, stretching, or meditating	0.92 (0.37–2.28) *P* = .860	Higher income 4.10 (1.46–11.45) *P* = .007
Engaging in healthy behaviors like trying to eat healthy, well-balanced meals, exercising regularly, getting plenty of sleep, or avoiding alcohol and drugs	0.32 (0.14–0.72) *P* = .006	
Making time to relax	0.39 (0.17–0.90) *P* = .027	
Connecting with others, including talking with people you trust about your concerns and how you are feeling	0.30 (0.13–0.68) *P* = .004	Higher income 2.37 (1.01–5.55) *P* = .047
Contacting a healthcare provider	0.41 (0.14–1.20) *P* = .103	
Eating high fat or sugary foods	0.22 (0.09–0.54) *P* = .001	
Eating more food than usual	0.23 (0.08–0.65) *P* = .006	Higher income 4.74 (1.14–19.73) *P* = .033
Eating less food than usual	1.44 (0.29–7.27) *P* = .659	

Note. *CI* confidence interval

^a^Includes all women who identified as Hispanic, Black, Asian, or multiple races

#### Demographic disparities

Women of color (i.e., women who identified as Hispanic, Black, Asian, or multiple race) and women with lower incomes were more likely to report serious financial problems compared to non-Hispanic White women (OR = 0.20, 95%CI = 0.05–0.87) and women with higher incomes (OR = 0.27, 95%CI = 0.08–0.93) (Table 2). Women with lower incomes were also less likely to engage in healthy stress-coping behaviors compared to women with higher incomes, such as taking care of their bodies (OR = 4.10, 95%CI = 1.46–11.45) and connecting with others during the pandemic (OR = 2.37, 95%CI = 1.01–5.55).

While mean scores on the ISEL and ISEL subscales indicated high levels of social support (total ISEL M = 39.83; SD = 7.76; Appraisal Support M = 14.22, SD = 2.65; Belonging Support M = 12.18, SD = 3.21; Tangible Support M = 13.25, SD = 3.25), disparities also existed for interpersonal support (Table 3). Women with higher incomes (*b* = 0.30, *p* = .012), not on Medicaid (*b* = − 0.25, *p* = .034), and non-Hispanic White women (*b* = 0.29, *p* = .007) reported higher ISEL-total scores compared to women with lower incomes, women on Medicaid, and women of color, respectively. The same pattern of results was found for ISEL-appraisal support. Women with higher incomes (*b* = 0.34, *p* = .003), not on Medicaid (*b* = − 0.24, *p* = .037), and non-Hispanic White women (*b* = 0.24, *p* = .017) reported higher ISEL-appraisal support compared to women with lower incomes, women on Medicaid, and women of color. Non-Hispanic White women also reported higher ISEL-belonging support (*b* = 0.25, *p* = .028) and ISEL-tangible support (*b* = 0.38, *p* = .001) compared to women of color.

**Table 3:** Differences in interpersonal support in perinatal women

	Total ISEL	ISEL-Appraisal	ISEL-Belonging	ISEL-Tangible
Pregnant vs. postpartum	.04, *p* = 714	.01, *p* = .921	.02, *p* = .853	.12, *p* = .251
Age	.001, *p* = .995	.14, *p* = .187	−.03, *p* = .821	−.07, *p* = .537
Income	.30, *p* = .012	.34, *p* = .003	.19, *p* = .137	.22, *p* = .066
Medicaid status	−.25, *p* = .034	−.24, p = .037	−.21, *p* = .094	−.19, *p* = .113
Race/ethnicity	.29, *p* = .007	.24, *p* = .017	.25, *p* = .028	.38, *p* = .001

Note. Standardized regression coefficients are reported

### Qualitative content analysis

Content analysis codes and response rates, along with exemplar quotes describing the codes, are presented in Table 4.

**Table 4:** Qualitative content analysis codes, response rates, and exemplar quotes

	Pregnant (*n* = 42)	Postpartum (*N* = 37)	
**What are you most worried about regarding COVID-19 and your pregnancy? Or if you have already delivered your baby, what are you most worried about regarding COVID-19 and newly parenting?**			
Baby contracting COVID-19	52% 22/42	49% 18/37	“I am most worried that a wave of COVID-19 will be a risk to my baby after I deliver in October. I know that logically, newborns and infants are not a high-risk group if they contract COVID-19, but I still feel sick at the idea of my new baby at risk for the virus.” #119, pregnant “I am worried that the general consensus of ‘COVID doesn’t affect kids’ is totally wrong […] the disease is so new that we don’t really know for sure.” #142, postpartum
Self or partner contracting COVID-19	38% 16/42	32% 12/37	“I am worried about contracting COVID while pregnant. There are still so many unknowns and I am worried about it causing pregnancy complications.” #105, pregnant “I had a c-section and during that time I was worried that if I caught COVID my immune system wouldn’t be able to cope. I was afraid to die and leave my baby behind.” #126, postpartum “Not being able to take care of him if I were to get severely sick from COVID-19.” #148, postpartum “Me or my husband contracting the virus and giving it to our newborn.” #162, postpartum
Isolating from baby should mom or baby have COVID-19	17% (7/42)	14% (5/37)	“I am most worried about being separated from my baby when I deliver if I test positive and that’s the hospital’s policy.” #87, pregnant “That COVID will separate my baby from me.” #139, postpartum
Family/others missing key OB appointments & events	24% (10/42)	3% (1/37)	“A hospital having a policy that my husband cannot be in the delivery room, a local outbreak occurring so that my parents cannot safely visit the baby.” #104, pregnant
Lack of social support	2% 1/42	16% 6/37	“Not having support. As a first-time mom and trying to social distance means no help from friends and family.” #158, postpartum
Finances & resources	5% (2/42)	3% (1/37)	“I’m worried that my husband and I won’t have enough money to support our baby.” #97, pregnant “When I had my baby was when everyone was panic buying […] I could not get wipes, diapers, or a thermometer in store for a bit. People were also hoarding distilled water and formula which left me panicking that I wouldn’t have the items needed when baby came home.” #132, postpartum
New world for baby	0% (0/42)	3% (1/37)	“What kind of world I will be raising my baby in.” #159, postpartum “I’m most worried about the world around us changing so drastically that I can’t give my baby the life I had planned on.” #127, postpartum
**Are there things that you are lacking (food, diapers, etc.) that are making you feel stressed or anxious?**			
Cleaning and baby supplies	12% (5/42)	32% (12/37)	“I have had a hard time getting diapers, formula, wipes and other nursing/pumping supplies. In addition, many cleaning products are out of stock (everything from hand wash, dish soap to disinfectants) which are needed in greater quantities with a newborn.” #137, postpartum
Financial support	21% (9/42)	0% (0/37)	“My business has been closed since March 17th which has caused a lot of stress and a financial strain.” #88, pregnant
Social support	7% (3/42)	3% 1/37	“Not having my family to support me because we are isolating ourselves.” #95, pregnant
Childcare	2% (1/42)	3% (1/37)	“Yes, husband deployed, so lack of partner support and for childcare for my other child during appointments […]” #124, pregnant “Support groups, finances, support from work to care for child, safe childcare options or longer time to stay at home with child.” #134 postpartum
**What resources would be most helpful to you?**			
COVID-19 info	38% (16/42)	19% (7/37)	“More information concerning how to best protect myself and a newborn during a pandemic.” #116, pregnant “I wish to see more studies about pregnant women who contracted COVID-19. I would feel better if there was more information about what could possibly happen if I were to get COVID-19 while pregnant.” #119, pregnant “Medical resources and cases of how other newborns and infants have been affected if they are infected.” #149, postpartum
Financial assistance	14% (6/42)	5% (2/37)	“Unemployment has been so hard to get a hold of and their website crashes every time I try to apply because of traffic on the site. I wish there was a more local number or somewhere I could get financial help […].” #85, pregnant “Grants for small businesses. Right now we are only getting loans and minimal unemployment for my husband.” #88, pregnant
Healthcare access	36% (15/42)	35% (13/37)	“Resources for online classes that used to be in person - birth classes, hypnobirthing, breastfeeding.” #121, pregnant “I think the most helpful resource would be more lactation support or more support for postpartum depression during a pandemic.” #128, postpartum
Access to groceries and supplies	5% (2/42)	19% (7/37)	“Fair access to diapers, wipes, cleaning supplies.” #106, pregnant “Updated list of in stock items available on store websites.” #153, postpartum
Workplace needs	5% (2/42)	11% (4/37)	“More talking about risks we are facing and how to minimize risks, while at work. Especially when it comes to pregnancy and working in a health care environment.” #93, pregnant
Social support	10% (4/42)	16% (6/37)	“Online support group/chat for new parents in this time.” #113, postpartum “It would be nice to be able to have more support from family.” #109, pregnant
Childcare	2% (1/42)	8% (3/37)	“More affordable childcare options for essential workers that allow pre-enrollment meetings.” #146, postpartum “Family being able to help take care of baby.” #162, postpartum

Note. Percentages do not sum to 100% as participants were allowed to provide several answers to each question

#### Primary stressors

Participants reported being most worried about their baby contracting COVID-19, with 52% pregnant and 49% postpartum women reporting this concern. This was followed by their concern of self or partners contracting COVID-19 (38% pregnant and 32% postpartum participants noted this concern):

*“I had a c-section and during that time I was worried that if I caught COVID my immune system wouldn’t be able to cope. I was afraid to die and leave my baby behind.”* #126, postpartum.

These concerns were almost always connected to one another, as depicted here, as this participant notes their greatest concern during the pandemic:

*“Me or my husband contracting the virus and giving it to our newborn.”* #162, postpartum.

Participants were also concerned about isolating from their baby, should they or their infant test positive for COVID-19 (17% pregnant and 14% postpartum participants noted this concern). Other concerns noted were family missing key obstetric appointments (e.g., ultrasounds) and events (e.g., delivery), lack of social support, their baby entering “a new world,” and finances and resources.

“*I’m worried that my husband and I won’t have enough money to support our baby.”* #97, pregnant.

#### Items and resources lacking that are causing stress

Participants noted they were lacking cleaning and baby supplies (12% pregnant and 32% postpartum participants).

*“I have had a hard time getting diapers, formula, wipes and other nursing/pumping supplies.”* #137, postpartum.

As reported in the other qualitative codes, financial needs (12% pregnant participants), social support (7% pregnant and 3% postpartum participants), and childcare (2% pregnant and 3% postpartum participants) were also stated as lacking during the COVID-19 pandemic, and these were often described together, as reported here:

*“Support groups, finances, support from work to care for child, safe childcare options or longer time to stay at home with child.”* #134, postpartum.

#### Resources needed

Participants indicated that the primary resources they needed were information regarding COVID-19 (38% pregnant and 19% postpartum participants) and access to healthcare (36% pregnant and 35% postpartum participants):

*“More information concerning how to best protect myself and a newborn during a pandemic.”* #116, pregnant.

*“Resources for online classes that used to be in person - birth classes, hypnobirthing, breastfeeding.”* #121, pregnant.

This was often related to the need for social support (10% pregnant and 16% postpartum participants):

*“Online support group/chat for new parents in this time.”* #113, postpartum.

Financial assistance (14% pregnant and 5% postpartum participants), access to groceries and supplies (5% pregnant and 19% postpartum participants), workplace needs (5% pregnant and 11% postpartum participants), and childcare (2% pregnant and 8% postpartum participants) were also noted.

*“Fair access to diapers, wipes, cleaning supplies.”* #106, pregnant.

*“Updated list of in stock items available on store websites.”* #153, postpartum.

## Discussion

Our study found that food scarcity and shelter in place restrictions as a result of the COVID-19 pandemic have made it difficult for pregnant women to find healthy foods. The stress resulting from food scarcity implicates acutely-responsive systems like stress physiology, while also impacting intergenerational effects of the mother’s chronic nutrition adversity (including during pre-pregnancy) on offspring development [31]. Financial issues were also prevalent in our sample and participants had difficulty obtaining childcare. Our qualitative results support the survey findings as financial resources and access to healthcare were primary concerns and resources needed by participants.

While participants reported missing prenatal appointments, many also reported using telemedicine for prenatal appointments and talked to their provider about labor and delivery and COVID-19. This stressor corresponds with recent research indicating that pregnant women experienced elevated levels of stress related to feeling unprepared for birth during the pandemic [24, 25]. Telemedicine is a useful resource for perinatal women during the pandemic as it decreases waiting times and risk of infection by greatly limiting in-person clinic visits. Patient education regarding the use of this video service and following best practices for telemedicine (e.g., teaching patients how to download and use the video platform prior to their appointment, making sure the patient has strong connection to reliable internet [32]) will aid in decreasing patient stress. Unfortunately, there are barriers to the access of telemedicine as it may be difficult for patients to be connected to these services in remote or rural areas. Additionally, healthcare providers should acknowledge that not all perinatal women own or have access the necessary equipment to support video calls and the lack of resources can be a burden on women’s access to essential care [32].

To cope with social distancing, isolation, and stress during the pandemic, participants took breaks from news outlets, engaged in healthy behaviors such as trying to eat healthy, exercising, getting plenty of sleep, avoiding alcohol and drugs, connecting with others, and making time to relax. However, pregnant women were less likely to engage in these healthy stress-coping behaviors than postpartum women. It may be that pregnant women are less likely to engage in healthy stress coping behaviors because they are focused on self-isolating due to concerns about contracting COVID-19 and being separated from their newborn after delivery, compared to postpartum women. Conversely, postpartum women may feel more connected with others once their baby is born, and attention may shift to caring for their baby versus stress about COVID-related “what ifs”. Our qualitative results elaborate on these findings, as many pregnant women expressed concerns about their baby contracting COVID-19 while in the hospital, and significant others missing the delivery or key OB appointments, and wanting support from friends, family, and birthing classes. Our findings further corroborate recent research that indicates that there is high prevalence of stressors related to perinatal COVID-19 infection in pregnant women [24, 25].Additional research is needed to further explore differences in stressors and coping behaviors between pregnant and postpartum women.

While our sample was predominantly non-Hispanic White, we were able to detect signals of greater stress induced by the COVID-19 pandemic, and less social support, in perinatal women of racial and ethnic minority and lower-income status. Women of color and women with lower incomes were more likely to report serious financial problems compared to non-Hispanic White women and women with higher incomes. Women with lower incomes were also less likely to engage in healthy stress-coping behaviors compared to women with higher incomes. Women with higher incomes, not on Medicaid, and non-Hispanic White women reported more social support compared to women with lower incomes, women on Medicaid, and women of color. These differences highlight the need to examine the sociocultural contexts of psychological adaptation to perinatal stress and coping [16–18], which may be exacerbated during the COVID-19 pandemic. This is also in line with previous research noting women in lower socioeconomic groups have an increased risk for stress and pregnancy complications [22], recent research indicating that women of color experienced elevated levels of stress related to feeling unprepared for birth or being worried about perinatal infection [25].

Pandemic-related stress experienced by perinatal women underscore the need to mitigate the downstream adverse impacts of the COVID-19 pandemic on maternal-infant health. In particular, women in lower socioeconomic groups and women of color may have increased stress and stressors, and fewer resources to cope with their stress, adding to the disparity in COVID-19 diagnoses, hospitalization, clinical outcomes, and mortality [33–36]. Assessing perinatal stress and isolation as it relates to the COVID-19 pandemic, and increasing access to psychological services via telehealth, may be useful in preventing negative maternal-infant outcomes. Early mental health interventions may help lower the risk of postpartum depression and help promote the long-term wellbeing of mother and baby [37]. Additional resources that may help alleviate stress include assurance of safe access to healthcare, access to groceries and supplies, online social support via parenting groups, and financial assistance.

Limitations include the cross-sectional design and nationwide response, which presents a single snapshot of stressors and coping of American perinatal women during the COVID-19 pandemic. We expect that stress and coping may fluctuate depending on shelter-in-place orders, rates of COVID-19 confirmed cases, hospitalizations, and deaths, and access to crucial resources (health food, formula, diapers, etc.) in local communities. In addition, while some counties have been in a “Phase 1” shelter-in-place for over 6 months, some states have not placed any restrictions on residents. However, research on stress and coping of perinatal women during the COVID-19 pandemic is critically needed, as research with pregnant women in China demonstrate a risk for mental illness during the pandemic [23],and research with pregnant women in the United States demonstrate elevated levels of anxiety and stress in this population [24, 25]. Therefore, our cross-sectional report of stressors, coping behaviors, social support, and resources needed in relation to the COVID-19 pandemic in a sample of perinatal women in the United States extends current literature by assessing social support and resources needed, using a qualitative approach to describe the patient perspective, and including postpartum women. This offers healthcare providers additional insight needed to mitigate psychological stress that can have important effects on maternal and infant health [7]. Future research should assess partner support and psychological services used during pregnancy and postpartum as early mental health interventions may help promote wellbeing of mother and baby [37].

In summary, we identified key stressors affecting perinatal women during the COVID-19 epidemic. Our study identifies several key messages relevant for healthcare providers:

- Healthcare providers should familiarize themselves with local services near their clinics and hospitals to better assist perinatal women in finding resources in their community. Some services that should be offered include food banks, Women, Infant, and Children Nutrition Program, and assistance with formula, diapers, and other supplies.
- Patient education about how to cope with stress may be an effective way to help perinatal women engage in healthier options at home
- Healthcare providers should engage in conversations regarding the burden of finding childcare when schools and daycares are closed and continue asking women if their kids feel safe at home, and how the loss of school resources has impacted their family. These questions will help assess the support and resources that perinatal women may need
- Accommodations should be made for those who cannot participate in telemedicine due to lack of access or language barriers. For example, women in lower socioeconomic groups and rural residents may not have consistent access to the internet
- To better support perinatal women’s mental health, healthcare providers should refer patients to counseling services, virtual support groups, and consistently screen pregnant women for depression and anxiety

## Conclusion

Psychological stress experienced during pregnancy can have important effects on maternal and infant health. We assessed stressors, coping behaviors, and resources needed during the COVID-19 pandemic in a sample of perinatal women in the United States. We identified a myriad of stressors from financial issues, difficulties finding healthy food, to missing healthcare appointments. Importantly, we observed that pregnant women were less likely to engage in healthy stress-coping behaviors than postpartum women. Lastly, we were able to detect signals of greater stress induced by the COVID-19 pandemic, and less social support, in perinatal women with racial and ethnic minority and lower-income status. Qualitative results support our survey findings as participants expressed concerns about their baby contracting COVID-19 while in the hospital, significant others missing the delivery or key OB appointments, and wanting support from friends, family, and birthing classes. Our findings are relevant for current providers and will help to inform interventions to mitigate stressors in this vulnerable population.

## Supplementary Information

**Additional file 1.** Open-ended qualitative questions and survey items.

**Additional file 2: Supplementary Table.** Consolidated criteria for reporting qualitative studies (COREQ): 32-item checklist.

## Data Availability

A de-identified dataset of the quantitative survey responses analyzed during the current study are available from the corresponding author upon reasonable request.

## Abbreviations

CI: 95% confidence intervals (CI)
COVID-19: Coronavirus disease 2019
ISEL: Interpersonal Support Evaluation List Short Form
M: Mean
OR: Odds ratio
SD: Standard deviation
