# Supplementary material for: Stressors, coping, and resources needed during the COVID-19 pandemic in a sample of perinatal women
Source: BMC Pregnancy Childbirth. 2021 Mar 1;21:171. doi: 10.1186/s12884-021-03665-0 (PMC7920400; doi:10.1186/s12884-021-03665-0)
Supplement: Supplementary file 1 — Additional file 1. Open-ended qualitative questions and survey items. [file 12884_2021_3665_MOESM1_ESM.docx]

**Additional file 1: Open-ended qualitative questions and survey items**

Open Answer

1. What are you most worried about regarding COVID-19 and your pregnancy? Or if you have already delivered your baby, what are you most worried about regarding COVID-19 and newly parenting?
2. Are there things that you are lacking (food, diapers, etc.) that are making you feel stressed or anxious?
3. What resources would be most helpful to you?

Demographic Information

Age: ______

Race (select all that apply):

- American Indian or Alaska Native
- Asian
- Black or African American
- Native Hawaiian or Other Pacific Islander
- White

Ethnicity:

- Hispanic
- Non-Hispanic

Where do you currently live?

State: ________________

County (not country): _____________________

What is the highest level of education you have completed? Less than high school

- Some high school
- High school graduate/GED
- Some college but no degree
- Technical/vocational degree or associate degree
- College graduate (bachelor’s degree)
- Post graduate education (master’s or doctoral degree courses)

Select your status (select all that apply):

- Employed full time
- Employed part time
- Unemployed
- Homemaker/Stay-at-home mom
- Student
- Retired

What was your total household income before taxes during the past 12 months?

- Less than $25,000
- $25,000 to $34,999
- $35,000 to $49,999
- $50,000 to $74,999
- $75,000 to $99,999
- $100,000 to $149,999
- $150,000 to $199,999
- $200,000 or more

Are you covered by health insurance?

- - Yes, military
  - Yes, employer-sponsored
  - Yes, individual
  - Yes, Medicare
  - Yes, Medicaid or CHIP
  - Yes, other (please specify: )________________
  - No

Medical and Social History

Have you had a confirmed COVID-19 diagnosis or a positive coronavirus test?

- Yes
- No

Has another member of your household had a confirmed COVID-19 diagnosis or a positive coronavirus test?

- Yes
- No

Has a member of your family, outside of your household, had a confirmed COVID-19 diagnosis or a positive coronavirus test?

- Yes
- No

How many children currently live in your household? _____________

What is your pregnancy status:

- Pregnant
- Postpartum

[If pregnant]

Did your pregnancy begin prior to the COVID-19 pandemic?

- Yes
- No

[If postpartum]

Did you give birth to your most recent baby before or during the COVID-19 pandemic?

- Before
- During

Have you given birth to other children?

- Yes
- No

[If yes]

How many older siblings does your baby have? ______

Since the onset of the COVID-19 pandemic, have you been unable to purchase? (select all that apply)

- [If pregnant] Healthy foods to support your pregnancy due to financial hardship
- [If pregnant] Healthy foods to support your pregnancy due to scarcity in stores
- [If pregnant] Healthy foods to support your pregnancy due to shelter in place restrictions
- N/A I was able to purchase all of the above

Since the onset of the COVID-19 pandemic, have you experienced any of the following? (select all that apply)

- Missed any prenatal care appointments due to COVID-19
- Used telemedicine for any prenatal care appointments due to COVID-19
- Sought additional information about how COVID-19 is impacting the hospital you plan to deliver
- Talked to your provider about labor and delivery and COVID-19
- None of the above

How has the COVID-19 outbreak affected you in the past two weeks? (select all that apply)

- Worked remotely or from home more than you usually do
- Worked reduced hours
- Was not able to work
- Had difficulty arranging for childcare
- Income or pay has been reduced
- Have no income
- Was laid off or furloughed by employer
- Had serious financial problems
- Another household member has lost their job
- None of the above

In the past two weeks have you experienced the following as a result of COVID-19? (select all that apply)

- Not enough money to pay rent
- Not enough money to pay for gas
- Not enough money to pay for food
- Did not have a regular place to sleep or stay
- None of the above

To cope with social distancing, isolation, or stress related to COVID-19, are you doing any of the following?

- Taking breaks from watching, reading, or listening to news stories, including social media
- Taking care of your body, such as taking deep breaths, stretching, or meditating
- Engaging in healthy behaviors like trying to eat healthy, well-balanced meals, exercising regularly, getting plenty of sleep, or avoiding alcohol and drugs
- Making time to relax
- Connecting with others, including talking with people you trust about your concerns and how you are feeling
- Contacting a healthcare provider
- Using sleeping medications or sedatives/hypnotics
- Eating high fat or sugary foods
- Eating more food than usual
- Eating less food than usual
- None of the above

Interpersonal Support Evaluation List

This scale is made up of a list of statements each of which may or may not be true about you. For each statement, select “definitely true” if you are sure it is true about you and “probably true” if you think it is true but are not absolutely certain. Similarly, you should check “definitely false” if you are sure the statement is false and “probably false” is you think it is false but are not absolutely certain.

1. If I wanted to go on a trip for a day (for example, to the country or mountains), I have a hard time finding someone to go with me.

- Definitely false
- Probably false
- Probably true
- Definitely true

2. I feel that there is no one I can share my most private worries and fears with.

- Definitely false
- Probably false
- Probably true
- Definitely true

3. If I were sick, I could easily find someone to help me with my daily chores.

- Definitely false
- Probably false
- Probably true
- Definitely true

4. There is someone I can turn to for advice about handling problems with my family.

- Definitely false
- Probably false
- Probably true
- Definitely true

5. If I decide one afternoon that I would like to go to a movie that evening, I could easily find someone to go with me.

- Definitely false
- Probably false
- Probably true
- Definitely true

6. When I need suggestions on how to deal with a personal problem, I know someone I can turn to.

- Definitely false
- Probably false
- Probably true
- Definitely true

7. I don't often get invited to do things with others.

- Definitely false
- Probably false
- Probably true
- Definitely true

8. If I had to go out of town for a few weeks, it would be difficult to find someone who would look after my house or apartment (the plants, pets, garden, etc).

- Definitely false
- Probably false
- Probably true
- Definitely true

9. If I wanted to have lunch with someone, I could easily find someone to join me.

- Definitely false
- Probably false
- Probably true
- Definitely true

10. If I was stranded 10 miles from home, there is someone I could call who could come and get me.

- Definitely false
- Probably false
- Probably true
- Definitely true

11. If a family crisis arose, it would be difficult to find someone who could give me good advice about how to handle it.

- Definitely false
- Probably false
- Probably true
- Definitely true

12. If I needed some help in moving to a new house or apartment, I would have a hard time finding someone to help me.

- Definitely false
- Probably false
- Probably true
- Definitely true
