# Supplementary material for: Stressors, coping, and resources needed during the COVID-19 pandemic in a sample of perinatal women
Source: BMC Pregnancy Childbirth. 2021 Mar 1;21:171. doi: 10.1186/s12884-021-03665-0 (PMC7920400; doi:10.1186/s12884-021-03665-0)
Supplement: Supplementary file 2 — Additional file 2: Supplementary Table. Consolidated criteria for reporting qualitative studies (COREQ): 32-item checklist. [file 12884_2021_3665_MOESM2_ESM.docx]

| **Supplementary Table.** Consolidated criteria for reporting qualitative studies (COREQ): 32-item checklist | |
| --- | --- |
| **Domain 1: Research team and reflexivity** |  |
| *Personal characteristics* |  |
| 1. Interviewer/facilitator | N/A. Qualitative data were collected via open-ended survey questions. There was no interviewer/facilitator. |
| 1. Credentials | Researcher credentials; occupation; gender; experience and training (personal characteristics #3-#5):  Celestina Barbosa-Leiker; PhD; Associate Professor, Washington State University (WSU) College of Nursing; Vice Chancellor for Research, WSU Health Sciences Spokane; female   - Stress in vulnerable populations; perinatal women with substance use disorders - Qualitative content analysis; Quantitative analyses; Mixed methods   Crystal Lederhos Smith; PhD; Assistant Research Professor, WSU Elson S. Floyd College of Medicine; female   - Substance use and co-use in high-risk populations - Trained and published in various forms of qualitative analysis including content analysis, phenomenological analysis, and grounded theory   Erica Crespi; PhD; Associate Professor, WSU School of Biological Sciences; female   - Stress physiology and endocrinology, Endocrine mechanisms of developmental programming   Olivia Brooks; MS; Research Associate, WSU College of Nursing and WSU Health Sciences Spokane; female   - Research coordination /management; substance use in high-risk populations; substance use and pregnancy - Qualitative content analysis   Ekaterina Burduli; PhD; Assistant Professor, WSU College of Nursing; female   - Maternal and newborn birth outcomes perinatal women with substance use disorders   Samantha Ranjo; student, WSU College of Nursing; female   - Nursing student   Cara Carty; PhD; Assistant Research Professor, Institute for Research and Education to Advance Community Health (IREACH), WSU Elson S. Floyd College of Medicine; female   - Maternal and child health; study design and data analysis   Luciana Hebert; PhD; Assistant Research Professor, IREACH, WSU Elson S. Floyd College of Medicine; female   - Reproductive, and women’s health; maternal and child health; quantitative data analysis   Sara Waters; PhD; Associate Professor, Department of Human Development, WSU Vancouver; female   - Biological and relational determinants of infant self-regulation development; Parent-child biological stress transmission   Maria Gartstein; PhD; Professor, Director of Clinical Training, Department of Psychology, WSU College of Arts and Sciences; female   - Infant reactivity and regulation; biological and contextual determinants of infant temperament   (General author information found on the title page, page 1, lines 9-28, and also detailed in the informed consent, page 6 lines 31-34). |
| 1. Occupation |  |
| 1. Gender |  |
| 1. Experience and training |  |
| *Relationship with participants* |  |
| 1. Relationship established | No relationship with participants was established prior to study commencement, or during the study. |
| 1. Participant knowledge of the interviewer | Prospective participants had knowledge of the investigators insofar as they were provided with the purpose of the study in the recruitment advertisement. The purpose as stated is, “to help understand the mental and behavioral health and related behaviors of pregnant women during the COVID-19 pandemic and how they change over time.” Eligible participants also were provided a list of investigators within the consent form which detailed their name, credentials, contact information and study role. There was no interviewer, as qualitative data was collected via open-ended questions within the survey (Page 6 lines 31-34). |
| 1. Interviewer characteristics | As there were no interviewers, characteristics of the investigators were not reported to participants. |
| **Domain 2: Study design** |  |
| *Theoretical framework* |  |
| 1. Methodological orientation & Theory | Qualitative content analysis using an inductive approach was used to analyze responses to open-ended questions. (Page 8 lines 37-44, page 9 lines 45-47) |
| *Participant selection* |  |
| 1. Sampling | Cluster sampling was done to identify pregnant or postpartum women residing the United States, who were ≥18 years of age, English-speaking, currently pregnant or had given birth recently (9/1/2019 through time of study). (Page 6 lines 4-12, page 8 lines 37-44) |
| 1. Method of approach | Women were directed to online surveys using social media and a crowdsourcing platform, Prolific ([www.prolific.co](http://www.prolific.com)). Inclusion criteria were ≥18 years of age, English-speaking, currently pregnant or had given birth between 9/1/2019-5/31/2020. Those who met inclusion criteria were provided with an informed consent form that they could download or email to themselves. Participants provided consent by clicking an electronic statement indicating that they reviewed the consent form and agreed to participate in the study. The Washington State University IRB approved this study as exempt from review. (Page 6 lines 19-34, page 9 lines 4-7) |
| 1. Sample size | N=162 (Page 6 lines 24-31) |
| 1. Non-participation | 228 people met inclusion criteria, of which 162 consented to participate in the study. (Page 6 lines 19-24) |
| *Setting* |  |
| 1. Setting of data collection | Online, via Prolific and Qualtrics survey platforms (Page 6 lines 4-7) |
| 1. Presence of non-participants | N/A. Researchers and non-participants were not present. |
| 1. Description of sample | Participants were 162 perinatal (125 pregnant and 37 postpartum) women aged 19-45 years (Mean = 31 ±4.8 SD); 79% non-Hispanic White, 7% Hispanic/Latino, 5% Black, 4% Asian, and 5% more than one race. Eighty-eight percent of the sample were employed, homemaker, or a student, 96% were covered by health insurance, 19% had state/federally funded insurance, and 56% had a total household income ≥$75,000/year. No participants were positive for COVD-19; one participant had a family member test positive for COVID-19 (Page 10 lines 38-60)  The subset of perinatal participants that was given additional items related to social support, stressors, and resources needed included 79 women (42 pregnant and 37 postpartum) women aged 23-42 years (Mean = 32, SD=4.0); 88% non-Hispanic White, 8% Hispanic/Latino, 1% Asian, and 3% more than one race. All participants in the subsample were employed, homemaker, or a student, 99% were covered by health insurance, 8% had state/federally-funded insurance, and 72% had a total household income ≥$75,000/year. Fifty-seven percent of pregnant women had no children in their household, with the remaining participants reporting 1-3 children in their household (M=.64, SD=.85); postpartum women had a range of 1-5 children in their household (M=1.65, SD=.85) (Page 11 lines 4-24) |
| *Data collection* |  |
| 1. Interview guide | The following open-ended questions were included in the survey:   - What are you most worried about regarding COVID-19 and your pregnancy, or if you have already delivered your baby, what are you most worried about regarding COVID-19 and newly parenting? - Are there things that you are lacking (food, diapers, etc.) that are making you feel stressed or anxious? - What resources would be most helpful to you?   (Page 9 lines 45-57) |
| 1. Repeat interviews | N/A. There were no interviews or repeat interviews conducted in this study design. |
| 1. Audio/visual recording | N/A. Qualitative data were collected via participants’ responses to open-ended survey questions. There was no audio or visual recording. |
| 1. Field notes | Field notes were taken at coder analytic meetings and at the various iterations of coding conducted to reduce the data into themes. As noted in the manuscript, investigators are sharing quantitative data. Qualitative data will not be shared. (Page 10 lines 4-7, page 21 lines 12-17) |
| 1. Duration | Eighty-three of the 162 participants were administered a 10-minute quantitative survey (recruited via crowdsourcing platform). Seventy-nine of 162 participants were administered a 25-minute survey (recruited via social media), which included qualitative questions and a social support survey. (Pages 8 line 57, page 9 line 4) |
| 1. Data saturation | Data saturation was discussed with the data coding team. Upon first meeting to discuss data coding, all coders agreed that saturation was reached based on frequency of consistent data supporting each theme, with no new data offering additional unique information. (Page 10 lines 19-27) |
| 1. Transcripts returned | N/A. Participants’ typed their unique responses to the open-ended questions included in the online survey, therefore there was no need to transcribe and return transcripts. |
| **Domain 3: Analysis and findings** |  |
| *Data analysis* |  |
| 1. Number of data coders | Three (Barbosa-Leiker, Brooks, Smith) (Page 10 lines 14-17) |
| 1. Description of the coding tree | Similar to a coding tree, we coded by levels using multiple, mutually exclusive categories, reducing categories to the finalized themes. (Page 10 lines 9-12) |
| 1. Derivation of themes | Themes were derived from the data. For each participant response, themes were generated for the words and phrases expressed by the respondents (implicit and explicit). Themes were then coded using a set of mutually exclusive categories (i.e., concepts). We then assessed frequency of each concept. Conceptual content analysis was completed by two coders and then verified by a third coder. (Page 10 lines 9-19) |
| 1. Software | IBM® SPSS was used to manage survey data. (Page 10 lines 26-29) |
| 1. Participant checking | Participants did not provide feedback on the findings. |
| *Reporting* |  |
| 1. Quotations presented | Participants quotations were presented to illustrate themes/findings. Each quotation was identified by a number. Confidential information is not identifiable by participant identification number. (Page 14 lines 4-48, page 15 lines 4-49, Table 4 pages 28-30) |
| 1. Data and findings consistent | Data is consistent with the findings presented. As this is a mixed methods study, quantitative and qualitative data are mixed in the interpretation and presentation of findings. (Page 15 line 54-59, page 16 lines 4-14 and 51-58, page 17 lines 4-31) |
| 1. Clarity of major themes | Themes were clearly presented in the findings. (Page 13 line 47, page 14 line 30, page 15 line 9, Table 4 pages 28-30) |
| 1. Clarity of minor themes | A description of minor themes is included in the results section. For example, under Resources Needed, minor themes included access to healthcare, social support, financial assistance, etc. (Page 13 lines 49-57, page 14 lines 12-14, 21-31, and 51-58) |
